# Editorial for the Fourth Issue

**Published:** 2011-04-30

**Authors:** Shraga Blazer

Dear Colleagues,

We are delighted to announce that the fourth issue of the new peer-reviewed open-access journal *Rambam Maimonides Medical Journal (RMMJ)* has now been published online at www.rmmj.org.il.

This Journal, while sponsored by Rambam Medical Center, represents an international initiative with active participation of leading international figures in medicine, biomedical science, and the ethical and philosophical aspects of health care. *RMMJ* is freely accessible via the Internet for immediate worldwide, open access to the full text of articles and fulfills the terms of the Creative Commons attribution license under which everyone is free to copy, distribute, and display the work, free of charge, for non-commercial personal and educational purposes.

The editorial policy of *RMMJ* is guided by the highest standards of scientific quality and integrity, professional responsibility, and human compassion in accordance with the Rambam’s (Maimonides) scholarly and ethical legacy.

As you may have noticed in the previous issues, *RMMJ* focuses on a wide spectrum of clinically relevant biomedical research and other issues central to the provision of optimal health care, disease prevention, and environmental health and provides a vehicle for original articles on basic and translational research that bring discoveries from the bench to the bedside and from the bedside to the community.

The present issue contains our regular sections “Nobel Laureate Perspective”, “The Maimonides (Rambam) Heritage”, and the “Rambam Grand Rounds”. The last-mentioned includes a special article of major current relevance on treatment of crush injuries caused by earthquakes. The “Rambam Forum” section deals with the current issue of the societal and religious considerations in the practice of organ donation. Two articles on major findings regarding diabetes are included in the “Horizons in Diabetes and Metabolism” section. Two new sections have been added, “Education, Practice and Organization of HealthCare in the 21st Century” and “Readers’ Opinions and Comments”, in which readers’ full-length articles that have been submitted as a response to an article previously published in *RMMJ* will be published along with the original article author’s reply. This kind of extended “Letter to the Editor” will enable an appropriate and comprehensive public discourse regarding various issues.

During the nine months that have passed since the inauguration, the *Journal* has had an outstanding exposure demonstrated by more than 11,000 new visits, and 59,000 articles downloaded, from 109 countries and territories. The *Journal* is a CrossRef member, and articles are indexed by Google Scholar and the Directory of Open Access Journals.

We call upon you to join us in this endeavor and cordially invite you to submit a paper to *RMMJ.* We would be grateful if you could also assist us by notifying your friends about the *Journal.* This can be easily done through our home website, by clicking the “Notify a Friend” button, or by sending us your friends’ email addresses through the “Contact Us” section.

With your help, the scientific and clinical excellence of the *Journal* will be assured.

Sincerely yours,

Shraga Blazer, MD Editor-in-Chief On behalf of the Editors

